# Artificial Intelligence for Procedural Guidance of Spinal Epidural Steroid Injections: A Scoping Review

**DOI:** 10.7759/cureus.108260

**Published:** 2026-05-04

**Authors:** Aleya DeVries, Taylor Yuska, Suzanne I Riskin

**Affiliations:** 1 Department of Foundational Sciences, Nova Southeastern University Dr. Kiran C. Patel College of Osteopathic Medicine, Clearwater, USA

**Keywords:** artificial intelligence, chronic low back pain, convolutional neural network, deep learning, herniated lumbar disc, lumbar spinal stenosis, lumbosacral radicular pain, magnetic resonance imaging, outcome prediction, transforaminal epidural steroid injection

## Abstract

Spinal epidural steroid injections (SESI) are commonly used for radiating low back and cervical pain but remain limited by target imprecision, operator variability, and inconsistent outcomes. Artificial intelligence (AI) has been increasingly investigated as a tool to augment image interpretation, trajectory planning, and decision support in interventional procedures, prompting interest in its potential role in procedural guidance for SESI. This scoping review aimed to summarize the current evidence on the use of AI to support procedural guidance for SESI.

A search was conducted across three databases, PubMed (National Library of Medicine (NLM)), Web of Science, and Embase, for English-language primary studies published between 2015 and 2025. Nine studies met the inclusion criteria. The AI architectures included machine learning, deep learning, and neural network-based models. Evidence was clustered into three domains: pre-procedural planning  (e.g., anatomic segmentation and trajectory optimization using CT or MRI), intraprocedural guidance (e.g., real-time needle localization or plane identification using ultrasound or robotic systems), and post-procedural outcome prediction (e.g., forecasting pain reduction following SESI based on imaging data).

Most studies demonstrated improvements in trajectory planning, needle localization, segmentation efficiency, or predictive performance. Potential benefits included reduced puncture time, decreased radiation exposure, and improved patient selection. However, AI performance was model-dependent, with some systems showing higher error rates than others. Additional limitations included small datasets, heterogeneity in AI model design, and underrepresentation of patients with spinal pathology or elevated body mass index. Overall, AI shows promise for enhancing SESI procedural guidance, but larger, more standardized studies are needed before its routine clinical use.

## Introduction and background

Low back pain (LBP) is the leading cause of years lived with disability (YLD), affecting an estimated 619 million people worldwide, and its prevalence is expected to increase substantially by 2050 [1]. Neck pain affects more than 200 million people globally and, like LBP, follows a similarly increasing trend in prevalence [2]. Both conditions are profoundly debilitating, impacting multiple domains of daily functioning and significantly reducing quality of life [3].

Common causes of chronic LBP and cervical pain include herniated discs, spinal stenosis, and degenerative disc disease [4]. However, across multiple specialties that manage LBP, including primary care, anesthesiology, and pain management, 90% of patients with LBP lack a definitive diagnosis for the cause of their pain [5]. First-line management of LBP typically includes nonprescription analgesics; however, additional pharmacotherapies, including antidepressants, muscle relaxants, and opioids, may be utilized depending on clinical context [6]. 

Some surgical interventions have been found to be superior to conservative management, especially invasive discectomy procedures [7]. Conversely, spinal epidural steroid injections (SESI) are a minimally invasive treatment approach that significantly reduce symptoms of sciatica and disability, and only 14% of SESI patients managed by a clinical pain management and interventional spine practice required surgical discectomy due to refractory pain [8].

The standard protocol for SESI, developed by the American Society of Interventional Pain Physicians' comprehensive evidence-based guidelines, involves the use of fluoroscopy-guided needle placement followed by corticosteroid injection into the epidural space [9,10]. The procedure is limited due to lack of target specificity, inconsistent post-procedural outcomes, and patient exposure to ionizing radiation [11]. The SESI also demonstrates a variable long-term success rate; although one study recorded initial improvement after six months, 77% of patients had recurrent pain four years after the initial SESI [12]. Due to these inconsistencies in treatment response, research efforts have increasingly focused on novel techniques and technologies to improve the efficacy of SESI, particularly in the areas of pre-procedural planning, intraoperative procedural accuracy, and prediction of post-procedural outcomes. 

Artificial intelligence (AI) consists of algorithms that mimic human cognitive processes to perform tasks such as recognition, classification, and prediction, making it increasingly relevant to clinical medicine [13]. There is substantial evidence for the use of AI in classifying medical images for the successful diagnosis of lung, skin, and brain diseases, as well as generating precise and reliable medical image segmentations and improving clinician efficiency [14,15]. Although the use of AI in surgical procedures remains in preliminary stages, deep learning AI models have demonstrated utility in guiding laparoscopic cholecystectomy procedures through identifying safe and dangerous zones of dissection and other anatomical structures in the surgical field [16]. With growing evidence supporting the usefulness of AI in detecting disease, improving clinician efficiency, and improving patient safety, an important question emerges: how can AI be applied to assist in improving SESI procedures? This study’s objective is to provide a scoping review of the current evidence for AI, including all software capable of learning and adapting to data sets, as a method of SESI procedural guidance and to recognize prospective challenges associated with its implementation.

## Review

Methods 

Eligibility Criteria 

This review was conducted in accordance with the Preferred Reporting Items for Systematic Reviews and Meta-Analyses Extension for Scoping Reviews (PRISMA-ScR) guidelines [17]. A formal review protocol was not prospectively registered, as protocol registration is not required for scoping reviews. This review included primary experimental studies (including randomized controlled trials (RCTs) and non-RCTs) and observational studies (including analytical observational studies, case-control studies, and analytical cross-sectional studies) published in English between 2015 and 2025. Systematic reviews and scoping reviews were excluded. Eligible studies involved SESI in patients or anatomical/phantom models in which AI was used for procedural guidance. Studies were excluded if the population was not undergoing SESI or if AI was not used for procedural guidance. Certain studies that used intraoperative robotics or traditional data-driven prediction models were excluded due to non-AI-guided interfaces.

Search 

The review question, "What is currently known about the use of AI for the procedural guidance of epidural spinal injections in clinical or experimental studies conducted in patients or models?” guided the search. The studies were collected through screening the electronic databases PubMed (National Library of Medicine (NLM)), Web of Science, and Embase, including English-language papers published between 2015 and 2025. The search strategy combined keyword terms related to epidural steroid injections (epidural steroid injection, transforaminal epidural, interlaminar epidural, and caudal epidural) and AI (artificial intelligence, machine learning, deep learning, and neural network). An example search string done in PubMed is provided in Table 1. Search strategies were adapted for each database to reflect differences in indexing and search interfaces. 

**Table 1:** Search string used for article retrieval

Search	Query	Results
#1	(((((("2015/01/01"[Date - Publication] : "3000"[Date - Publication])) AND (artificial intelligence[Title/Abstract])) OR (epidural steroid injection[Title/Abstract])) OR (Transforaminal epidural[Title/Abstract])) OR (caudal epidural[Title/Abstract])) OR (SESI[Title/Abstract])	108,241
#2	((((((epidural steroid injection) OR (Transforaminal epidural steroid injection)) OR (caudal epidural steroid injection)) OR (SESI))) AND ((((((("2015/01/01"[Date - Publication] : "3000"[Date - Publication])) AND (artificial intelligence[Title/Abstract])) OR (epidural steroid injection[Title/Abstract])) OR (Transforaminal epidural[Title/Abstract])) OR (caudal epidural[Title/Abstract])) OR (SESI[Title/Abstract]))) NOT (Systematic Review[Title/Abstract])	1,769
#3	(((((((((epidural steroid injection) OR (Transforaminal epidural steroid injection)) OR (caudal epidural steroid injection)) OR (SESI))) AND ((((((("2015/01/01"[Date - Publication] : "3000"[Date - Publication])) AND (artificial intelligence[Title/Abstract])) OR (epidural steroid injection[Title/Abstract])) OR (Transforaminal epidural[Title/Abstract])) OR (caudal epidural[Title/Abstract])) OR (SESI[Title/Abstract]))) NOT (Systematic Review[Title/Abstract])) NOT (systematic review)) NOT (review)) NOT (guidelines)	1,462
#4	( "artificial intelligence"[Title/Abstract] OR "machine learning"[Title/Abstract] OR "deep learning"[Title/Abstract] OR "neural network*"[Title/Abstract] ) AND ( "epidural steroid injection"[Title/Abstract] OR "epidural injection*"[Title/Abstract] OR "epidural corticosteroid injection"[Title/Abstract] OR "epidural corticosteroid"[Title/Abstract] OR "transforaminal epidural steroid injection"[Title/Abstract] OR "transforaminal injection*"[Title/Abstract] OR "caudal epidural injection*"[Title/Abstract] OR "interlaminar epidural injection*"[Title/Abstract] OR "spinal injection*"[Title/Abstract] OR "lumbar injection*"[Title/Abstract] OR "facet joint injection*"[Title/Abstract] OR "TFESI"[Title/Abstract] OR "ESI"[Title/Abstract] ) AND ("2015/01/01"[Date - Publication] : "3000"[Date - Publication])	167

Selection and Sources of Evidence 

**Figure 1:**
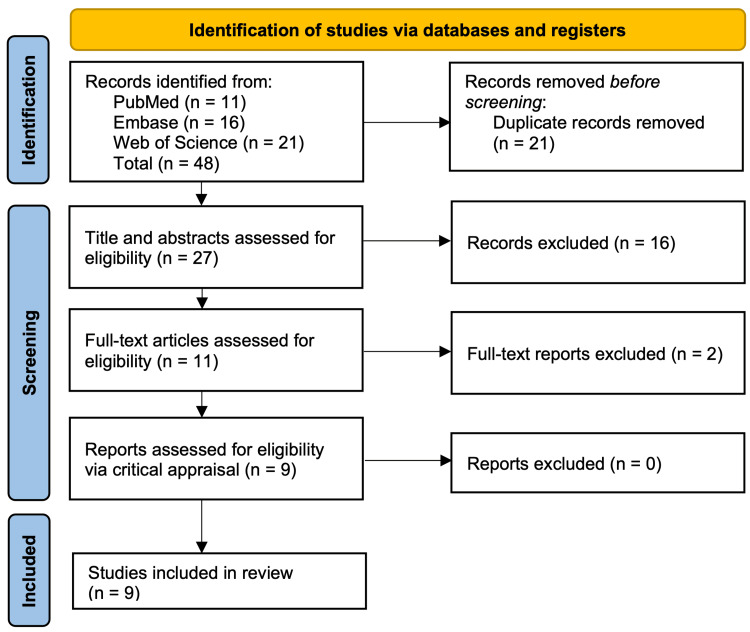
The PRISMA-ScR diagram PRISMA-ScR: Preferred Reporting Items for Systematic Reviews and Meta-Analyses Extension for Systematic Reviews [17]

The study selection was completed by screening abstracts and full-text articles against the inclusion criteria. The study selection process conducted per the Preferred Reporting Items for Systematic Reviews and Meta-Analyses Extension for Scoping Reviews (PRISMA-ScR) guidelines [17] is captured in Figure 1. Data from the final nine studies were extracted and charted in Microsoft Excel (Microsoft Corp., Redmond, WA, USA). The extracted data included the author, year of publication, purpose, study design, population, and sample size, methods, limitations, and key findings. Due to the heterogeneity in study design, AI models, and reporting metrics, a quantitative synthesis was not appropriate. Therefore, a descriptive synthesis approach was used to summarize study characteristics and key findings. 

Data Extraction, Synthesis, and Analysis

A data extraction table was developed to capture key study characteristics, including author, year, purpose, study design, population, methods, limitations, and key findings. Data charting focused on the qualitative descriptions of how AI was used in the context of SEIS. Quantitative performance of the AI modality was also extracted and reported where available. Data were extracted by a single reviewer and verified by a second. A critical appraisal and a risk of bias assessment of the included studies were not conducted, as the aim of this scoping review was to map the existing literature rather than assess the quality of evidence. The charted data were analyzed for key themes regarding the application of AI and categorized based on how AI was applied within the context of SESI into three categories: outcome prediction, pre-procedural planning, and procedural accuracy.

Results 

**Figure 2:**
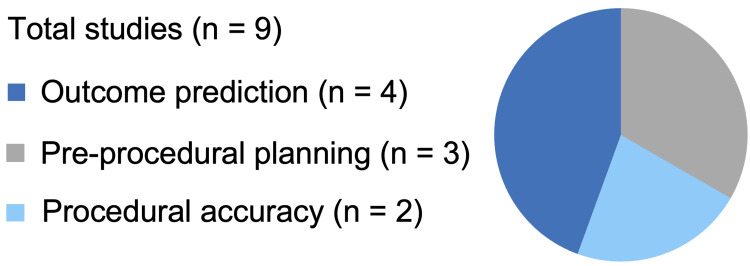
The distribution of studies across key themes summarized in a pie chart Outcome prediction: 44.4% of studies showed evidence of AI use in outcome prediction for SESI (n=4) [20,22,23,25]. Pre-procedural planning: 33.3% of studies demonstrated significant results of AI utilization for preoperative assessment (n=3) [19,24,26]. Procedural accuracy: 22.2% of studies demonstrated significant results of AI used for reliable procedural execution (n=2) [18,21]. SESI: Spinal epidural steroid injections

Based on the data collected, this scoping review qualitatively mapped how AI has been used in SESI procedural guidance. In total, nine studies met the inclusion criteria for this scoping review. The evidence was categorized into three domains: pre-procedural planning, procedural accuracy, and post-procedural outcomes. Table 2 summarizes the included studies. Additionally, a pie chart (Figure 2) visually summarizes the distribution of key themes across the nine included studies.

**Table 2:** Summary of the included studies (total n = 9) ANN: Artificial neural network, AUC: Area under curve, BNN: Bayesian neural network, CNN: Convolutional neural network, NRS: Numeric rating scale, TFESI: Transforaminal epidural steroid injection, 3D nnUNet: 3D-no-new-U-net

Author and year of publication	Objective	Study design	Population	Methods	Limitations	Key findings
Pesteie et al., 2015 [18]	To assess the feasibility of whether a real-time machine learning AI system could recognize the optimal plane in ultrasound for epidural and facet joint injections.	Experimental feasibility study	Healthy volunteers (n=13)	Assess the generalizability of machine learning, artificial neural network (ANN), trained to identify Hadamard features on ultrasound volumes and interpret target vs. non-target images.	Images were of healthy patients without spinal pathology or obesity.	AI with ultrasound classified epidural and facet joint injections planes with 95% and 94% accuracy, respectively, outperforming conventional filters.
Fan et al., 2019 [19]	To investigate the use of deep learning-based segmentation of lumbosacral nerves on CT to benefit the viability assessment of transforaminal epidural steroid injection (TFESI).	Translational imaging study	Lumbar CT scans from patients (n=50)	Retrospectively recruited lumbar spine radiographs were used to evaluate the lumbosacral segmentation accuracy of the deep learning model, 3D U-net spine CT, compared to manual segmentation.	More cases to confirm accuracy. Limited to L5/S1.	AI segmented spinal landmarks on CT images significantly faster than manual marking, with no significant difference in accuracy for anatomical landmarks.
Kim et al., 2022 [20]	To investigate the accuracy of a convolutional neural network (CNN) trained with lumbar MRI images for predicting therapeutic outcomes after TFESI.	Retrospective cohort study	Patients with lumbar radicular pain (n=503)	Retrospectively recruited lumbar spine radiographs labeled with post-TFESI pain reduction outcome scores trained a CNN to predict TFESI outcomes.	Limited training data.	CNN classified postoperative outcomes of lumbar TFESI with good discriminative power (area under the curve (AUC)= 0.827) and generalizability.
Lu et al., 2023 [21]	To evaluate the capability of a novel Bayesian neural network (BNN)-guided robot to assist in automatically maintaining the spinal epidural probe at the target tissue layer.	Experimental phantom study	Lumbar phantom spine models (10 trials)	Lumbar punctures and epidural steroid injection trials were performed, with bioimpedance needle insertion guided by a feedback control from a BNN algorithm.	Deformation of materials. The robot was not calibrated for respiratory motion.	An AI-guided robotic system identified tissue layers and maintained needle placement in the target tissue layer in eight out of 10 phantom experiments.
Wang et al., 2023 [22]	To assess the CNN-trained AI for cervical axial MRI to predict therapeutic outcome after cervical TFESI in patients with cervical foraminal stenosis.	Retrospective cohort study	Patients with cervical foraminal stenosis (n=288)	Retrospectively recruited cervical spine radiographs and MRI labeled with post-TFESI pain reduction outcome scores to train the CNN to predict TFESI outcomes. The performance of the CNN was tested with a set of validation images.	A limited number of cervical MRIs to train the CNN algorithm, lack of cross-validation using alternative approaches other than CNN-based methods.	CNN classified postoperative outcomes of cervical TFESI with good discriminative power (AUC= 0.802), and had a validation accuracy of 79.3%.
Kim et al., 2024 [23]	To predict therapeutic outcomes after lumbar TFESI in patients with lumbosacral radicular pain caused by lumbar spinal stenosis using a trained CNN model.	Retrospective observational study	Patients with lumbar spinal stenosis (n=193)	Retrospectively recruited lumbar spine radiographs labeled with post-TFESI outcome scores trained a CNN to predict TFESI outcomes.	A small sample size and self-reported numeric rating scale (NRS) data were used for output data.	The CNN model had high discriminative power at classifying favorable vs. non -favorable outcomes in lumbar TFESI and had high precision for unfavorable outcomes.
Liu et al., 2024 [24]	To evaluate the efficiency and accuracy of lumbar epidural steroid injection robots guided by an ANN, 3D no-new (nn)U-Net algorithm at locating fiducial markers in MRI and CT scans, when compared to a traditional Welzl’s algorithm.	Proof-of-concept imagine validation study	Lumbar CT and MRI images (small sample; exact number not specified)	3D nnU-Net algorithm's ability to detect the fiducial markers compared to theoretical values is measured with marking error evaluation and position error evaluation.	Insufficient supply of medical imaging data for training nnU-Net learning models.	AI nn-U-Net had higher marking errors than manual methods, showing variability across AI models.
Wang et al., 2024 [25]	To predict therapeutic outcomes after cervical TFESI in patients with chronic cervical radicular pain caused by cervical foraminal stenosis using a trained neural network CNN model.	Retrospective cohort study	Patients with cervical radicular pain (n=293)	Retrospectively recruited cervical spine radiographs labeled with post-TFESI outcome scores to train a CNN to predict TFESI outcomes.	Data from a single clinic was used, limiting the data set.	The CNN had high discriminative power at classifying favorable vs. non-favorable outcomes in cervical TFESI and had lower precision for unfavorable outcomes.
Su et al., 2025 [26]	To propose a preoperative planning method based on 3D AI-generated lumbar models to improve the accuracy and efficiency of the TFESI.	Experimental phantom study	Lumbar phantom models (n=13)	24 puncture trials were conducted on 3D lumbar phantom models generated using AI. Needle placement and puncture error were compared without pre-planned guidance.	No clinical trial verification, limited model sample size.	AI-generated 3D phantom models significantly reduced puncture time and puncture errors compared to conventional fluoroscopy.

Pre-procedural Planning 

Of the nine included studies, three described how AI can be used in the pre-procedural planning of SESI [19,24,26]. All three studies conducted pre-procedural planning specifically at the lumbar level of the spine [19,24,26]. One study used AI to generate a 3D phantom model based on patient data in order to conduct pre-procedural planning. In trials where the AI-generated 3D model was used in preoperative planning, there were significant reductions in the average SESI puncture time and puncture errors compared to those of conventional fluoroscopy [26].

Two of the three studies evaluated whether AI could interpret pre-procedural imaging for preoperative assessment as accurately as expert physicians [19]. This model segmented lumbar CT images significantly faster than manual segmentation, and its measurements of key anatomical landmarks did not differ significantly from manual measurements [19]. The second study using CT and MRI images demonstrated significantly higher marking error compared with both non-AI automatic geometric algorithms and manual marking [24].

Intraoperative Procedural Accuracy 

Of the nine final studies, two studies evaluated how AI could improve procedural accuracy or reduce reliance on radiation-based imaging during SESI [18,22]. Pesteie et al. investigated whether a machine learning model could identify injection planes using ultrasound volumes during SESI [18]. The study found that the model's extraction time per image was faster than conventional ultrasound local phase tensor (LPT) filters. The AI model correctly classified the target planes in 95% of cases for epidural injections and in 94% of cases for facet joint injections [18]. 

Lu et al. evaluated a robot-guided system powered by AI to assist with tissue layer identification during SESI [21]. The AI-guided robot was tested on a realistic phantom model and compared with manual fluoroscopy. In eight out of 10 preliminary experiments, the AI-guided robot successfully maintained the epidural probe at the correct tissue layer, without the need for continuous fluoroscopic confirmation [21]. 

*Post-procedural Predictive Capacity* 

Of the nine final studies, four studies investigated how AI can be utilized to predict post-procedural outcomes [20,22,25]. Two studies used retrospectively recruited MRI and two used retrospectively recruited radiographs [20,22]. Two studies focused on the post-transforaminal epidural steroid injection (TFESI) outcomes of the lumbar spine, and two focused on the post-TFESI outcomes of the cervical spine [20,22,23,25].

Both studies using plain radiographs to determine postoperative outcomes of lumbar and cervical TFESI found that the AI model had high discriminative power in classifying favorable vs. unfavorable outcomes [23,25]. The study conducted using cervical plain radiographs reported that the AI model had lower precision for unfavorable outcomes [25]. Conversely, the study conducted on the lumbar spine had higher precision for unfavorable outcomes [23]. Similar to the radiograph studies, AI models trained using T2-weighted MRI classified postoperative outcomes of lumbar and cervical TFESI with good discriminative power; however, the validation accuracy with the cervical MRI was higher than that of the lumbar MRI [20,22].

Discussion 

The studies included in this scoping review were assessed to summarize the utilization of AI in the procedural guidance of SESI. Additionally, this review categorized the current literature findings for the use of AI in SESI into three domains: pre-procedural planning, procedural accuracy, and post-procedural outcome prediction. The SESI carries risks, particularly exposure to radiation, which increases with puncture errors and puncture time because fluoroscopy is the traditional method used to confirm epidural space access and successful injection [10,27]. This scoping review summarizes preliminary results from an AI-generated phantom model that reported significant reductions in puncture time and puncture errors [26]. This finding suggests that using AI-generated phantom models may help reduce patient discomfort and radiation exposure while improving procedural accuracy. The 3D phantom models may also aid future medical training by helping less experienced clinicians reduce puncture time and error as they develop their technical skills. 

Comparison of AI Architectures in Pre-procedural SESI planning 

The performance of different AI architectures in pre-procedural planning varied notably across studies. The 3D U-Net AI system known as SPINECT, which has to be manually tuned to its data set, demonstrated strong potential for utility given its ability to segment CT images rapidly and with accuracy comparable to manual landmark measurement [19]. This suggests that semi-automated, clinician-tuned AI systems may meaningfully reduce pre-procedural workload without compromising precision. Conversely, the automatically adapting AI-based nnU-Net displayed a higher marking error compared to non-AI automatic geometric algorithms, and the system did not match the performance of manual marking [24]. The two architectures differ in that 3D U-Net AI requires manual image tuning, which may have contributed to its higher performance when compared to nnU-Net. These differences highlight that not all AI architectures are equally suited for SESI planning. Future research directly comparing architectures will be essential for determining the best approach.

Radiation Exposure 

Beyond pre-procedural planning, AI may also enhance procedural accuracy in ways that reduce reliance on fluoroscopy. Artificial neural network (ANN) models demonstrated the ability to identify injection planes using ultrasound volumes, offering a potential alternative to radiation-based imaging [18]. In a more novel application, a Bayesian neural network (BNN)-guided robot demonstrated the ability to identify and maintain the SESI probe at the correct tissue layer during phantom-model testing [21]. These preliminary findings suggest that both AI-assisted ultrasound and AI-guided robotic systems may help reduce radiation exposure to the patient without compromising the accuracy of the procedure.

AI-Based Prediction of SESI Outcomes and Patient Selection 

Previous literature has reported variable long-term outcomes following SESI [12]. The studies included in this review suggest that convolutional neural network (CNN)-based models demonstrate strong discriminative power in classifying favorable versus unfavorable outcomes regardless of radiographic or MRI modality [20,22,25]. These findings indicate that AI-generated predictions may help clinicians individualize pre-procedural counseling by identifying patients who are more or less likely to benefit from SESI. Improving patient selection is particularly important, as it may prevent patients with a low likelihood of benefit from undergoing unnecessary procedures. 

Although the results from these studies imply promise for AI application in procedural guidance of SESI, most studies remain preliminary, retrospective, or based on phantom models. The lack of clinical trial validation and small sample sizes reported in the literature make this area of study difficult to generalize. The AI architecture performance was also inconsistent across the studies, which makes it challenging to determine the most efficient and feasible applications of AI in the clinical setting. Together, these limitations make it difficult to ascertain the true potential of AI use for SESI. 

Future Directions for AI in SESI 

Future research may compare different AI architectures to determine the most suitable for SESI procedural guidance. Additionally, subsequent studies should investigate how to fully automate pre-procedural image marking without sacrificing accuracy. These applications of AI should be evaluated in more realistic clinical scenarios and larger sample sizes. Although the current evidence is compelling, substantial gaps remain, particularly in clinical validation and real-world feasibility.

## Conclusions

Spinal epidural steroid injections remain an important minimally invasive option for managing low back and cervical pain, yet their effectiveness varies widely. This scoping review summarizes the current evidence on the use of AI to enhance SESI procedural guidance, particularly in pre-procedural planning, procedural accuracy, and post-procedure outcome prediction. Collectively, early studies suggest that AI may improve procedural efficiency, enhance targeting accuracy, and support more individualized patient selection. However, most existing research is preliminary and limited by small sample sizes or nonclinical study designs. Larger studies and clinical trial validation will be essential to determine the feasibility and true clinical utility of AI-assisted SESI.
